# The emergence of NDM-1, IMP-7, IMP-26, IMP-62, IMP-76, VIM-4 and VIM-5 producing *Pseudomonas aeruginosa* strains, identification of a novel sequence type (ST3891) and analysis of virulence genes from a Malaysian tertiary hospital

**DOI:** 10.1371/journal.pone.0350200

**Published:** 2026-06-10

**Authors:** Kalaivani Kalai Chelvam, Yue Min Lim, Cindy Shuan Ju Teh, Sasheela Ponnampalavanar, Jamuna Vadivelu, Vanitha Mariappan, Kumutha Malar Vellasamy

**Affiliations:** 1 Department of Medical Microbiology, Faculty of Medicine, Universiti Malaya, Kuala Lumpur, Malaysia; 2 Department of Medicine, Faculty of Medicine, Universiti Malaya, Kuala Lumpur, Malaysia; 3 Dean Office, Faculty of Medicine, Universiti Malaya, Kuala Lumpur, Malaysia; 4 Center of Toxicology and Health Risk Studies (CORE), Faculty of Health Sciences, Universiti Kebangsaan Malaysia, Kuala Lumpur, Malaysia; Tribhuvan University, NEPAL

## Abstract

Multidrug-resistant (MDR) *Pseudomonas aeruginosa* is a medically crucial nosocomial pathogen causing significant mortality in patients with weakened immune systems. It is sensitive to carbapenems such as meropenem and imipenem. However, recent research indicates the rise and rapid transmission of carbapenem-resistant *P. aeruginosa* (CRPA). This study analysed the phenotypic and genotypic traits linked with CRPA strains obtained from the University Malaya Medical Centre (UMMC), a Malaysian tertiary teaching hospital. MDR *P. aeruginosa* (n = 215) were isolated from the Medical Microbiology Diagnostic Laboratory (MMDL). The VITEK-2 automated system was used for antimicrobial susceptibility and data for susceptibility to various antimicrobial agents were retrieved from the MMDL database at the UMMC. The minimum inhibitory concentration (MIC) of the carbapenems (meropenem and imipenem) was analysed using the broth microdilution method. Pyocyanin, protease and biofilm assays were conducted to assess the phenotypic traits. Furthermore, polymerase chain reaction (PCR) was used to detect six virulence genes and three carbapenemase genes. *P. aeruginosa* strains that displayed high-level resistance to meropenem and imipenem showed an association with 140 strains (65.1%) that were strong biofilm producers (OD_570nm_ ≥ 3.0). The majority of the strains (73.5%) exhibited high to moderate pyocyanin production, while 95.3% were capable of secreting protease. Among the six virulence genes tested, most of the strains harboured the *toxR* (109/215, 50.7%) and *algD* (108/215, 50.2%) genes. The identification of carbapenem genes showed *bla*_IMP_ (n = 24, 11.2%) followed by *bla*_NDM_ (n = 16, 7.4%) and *bla*_VIM_ (n = 4, 1.86%). A novel sequence type, ST3891 was discovered using multilocus sequence typing (MLST). The rising incidence of carbapenem-resistant strains is concerning, as carbapenems with antipseudomonal activity play a vital role in treating *P. aeruginosa* infections. Additionally, this study found a positive correlation between increased biofilm production and resistance to carbapenem in *P. aeruginosa.* This emphasises the importance of stringent infection control strategies, particularly considering the clonal dissemination of the high-risk ST235 clone and the identification of the novel ST3891 strain in Malaysia.

## Introduction

*Pseudomonas aeruginosa* related to multidrug resistance (MDR) is a medically crucial nosocomial pathogen causing significant mortality in patients with weakened immune systems. It is also one of the most frequently occurring bacterial co-infections in COVID-19 patients [1], with severe infections complicating their clinical management [1,2].

The Southeast Asian region recorded the highest history of *P. aeruginosa* prevalence [3]. It frequently affects patients with compromised immune systems [4]. Various studies have reported increasing isolation of MDR *P. aeruginosa* from hospitalised patients, especially in critically ill patients [5,6]. *P. aeruginosa* infections are generally treated with antibiotics. However, treatment is becoming more difficult due to increasing resistance to antibiotics.

In Malaysia, the National Surveillance of Antibiotic Resistance (NSAR) report indicated that both meropenem and imipenem showed an increasing trend in resistance until around 2014–2015, after which the resistance gradually declined up to 2018 [7]. By 2023, the resistance rates for meropenem and imipenem further decreased from 6.8% to 6.5% and 7.1% to 6.7%, respectively [8]. In University Malaya Medical Centre (UMMC), carbapenem-resistant *Enterobacteriaceae* (CRE), particularly *Klebsiella pneumoniae* was first reported in 2013, then peaked from 2014 to 2015 [9]. It continued to cause different infections among the patients from 2016 to 2017, leading to an increase in carbapenem-resistant *K. pneumoniae* (CRKP) infections [10]. Carbapenem-resistant *Acinetobacter baumannii* (CRAB) strains at UMMC exhibited resistance to most antibiotics, with colistin being the only effective option, highlighting limited treatment alternatives [11,12]. Data regarding the burden and underlying mechanisms of carbapenem-resistant *P. aeruginosa* (CRPA) in Malaysia is scarce, particularly when compared to information on CRKP and CRAB. Therefore, this study investigated phenotypic and genotypic characteristics associated with CRPA in UMMC. The potential benefits of this study include improved treatment strategies, better infection control measures and contributions to the broader scientific knowledge based on antibiotic resistance. This may positively influence patient care, public health and worldwide efforts against antibiotic resistance.

## Materials and methods

### Data collection

A retrospective analysis of archived bacterial cultures from clinical specimens were conducted. The data were accessed on 21 August 2020 for research purposes. Data involving patients’ demographics, including ethnicity, as well as the source of the specimen, location of the ward and antibiotic susceptibility were retrieved from the Medical Microbiology Diagnostic Laboratory (MMDL) database at University Malaya Medical Centre (UMMC), following institutional guidelines and ethical approval.

### Ethical considerations

The UMMC Medical Research Ethics Committee approved the ethics with the following reference number (MRECID.NO: 2019815−7748). Informed consent was not obtained. During data collection, the authors had access to identifiable information by using the patient web portal and written requests were required to access the patient data. After data extraction, all records were anonymised to protect patient confidentiality, in compliance with ethical and regulatory requirements.

### Bacterial strains

A total of 215 MDR *P. aeruginosa* strains from the MMDL collection from patients in UMMC from January 2015 to December 2018 were used in this study. MDR *P. aeruginosa* was defined as non-susceptible to at least one agent in ≥3 antimicrobial categories [13]. Non-MDR data were excluded from this study to focus on clinical relevance and resistance burden. The bacterial cultures were identified and confirmed via a polymerase chain reaction (PCR) assay (Table 1). *P. aeruginosa* ATCC 27853 was used as the positive control [14]. Strains PA153 and PACTR were isolated from the same patient three years apart. PA153 was recovered from bone in 2015 and PACTR was obtained from tissue in 2018.

**Table 1:** 16S rDNA-based primer set [14].

Primer	Sequence (5’ – 3’)	Annealing temp (°C)	Product size (bp)
PA-SS-F	GGGGGATCTTCGGACCTCA	58	956
PA-SS-R	TCCTTAGAGTGCCCACCCG		

### Antimicrobial susceptibility test (AST)

The antimicrobial susceptibility of the strains was evaluated using the AST-N314 card on the automated VITEK-2 system (BioMérieux, Marcy L’Etoile, France) by MMDL. Minimum inhibitory concentration (MIC) values of meropenem and imipenem (Oxoid, Hampshire, United Kingdom) were determined for each isolate using the broth microdilution method with concentrations ranging between 0 and 256 µg/mL, according to Clinical and Laboratory Standards Institute (CLSI) guidelines [15]. MIC breakpoints for meropenem and imipenem were established as ≤2 μg/mL for susceptible, 4 μg/mL for intermediate, and ≥8 μg/mL for resistant. *P. aeruginosa* ATCC 27853 was used as a quality control strain.

### Biofilm assay

Biofilm adherence assay was conducted using the crystal violet staining method as formerly outlined by Kalai Chelvam et al. [16] with slight modifications. The planktonic cultures (OD_600nm_ 1.0) were diluted in fresh Luria Bertani (LB) broth (Oxoid, UK) to 1:100 (~OD_600nm_ 0.01). Briefly, 100 μl of the culture was added into each well of a sterile 96-well plate and incubated at 37 °C for 24 hours. For the control wells, bacterial cultures were substituted with fresh LB. After incubation, the culture supernatant was aseptically transferred to a new sterile 96-well plate, and the absorbance at OD_600nm_ was measured to assess bacterial growth under biofilm conditions. The empty wells containing biofilm were stained with 150 μl of 1% crystal violet (w/v) and further incubated for 30 mins at room temperature. Excess stain was removed by washing the wells twice with 175 μl of sterile distilled water. Subsequently, 175 μl of dimethyl sulfoxide (DMSO) was added to each well, and after a 10-minute incubation at room temperature, the absorbance was measured at OD_570nm_. The quantification of biofilm formed (based on the OD_570nm_ of crystal violet stain) ranged between 0.01 and 5.71. The strains were divided into low (OD_570nm_ ≤ 1.5), moderate (OD_570nm_ 1.5 to 3) and high biofilm (OD_570nm_ ≥ 3) producers as suggested by published criteria [16]. The experiment was conducted in triplicate, with the mean OD_600nm_ value and its standard deviation (SD) reported.

### Protease assay

According to a protocol by García-Reyes, Moustafa [17], a skimmed milk agar was used to qualitatively determine the protease secretion. The inoculated plate was incubated for 48 hours at 37 °C. A clear zone around the colony becomes readily visible. The results were scored as no protease production – 0 (0 mm); low – 1 (≤ 1.5 mm; medium – 2 & 3 (1.5–3.0 mm); high – 4 (≥ 3 mm) based on the halo diameter [18]. The experiment was repeated twice to check for reproducibility. *P. aeruginosa* ATCC 27853 was used as the control strain.

### Pyocyanin assay

King A medium (comprising 20.0 g/L tryptone, 3.3 g/L MgCl₂·6H₂O, 20.0 g/L KOH, 5.5 mL H₂SO₄, and 10.0 g/L glycerol, adjusted to pH 7.2) [19] was inoculated with an overnight culture to an OD_600nm_ of 0.2 and incubated at 37 °C with constant shaking at 230 rpm. Following 24 hours of incubation, the medium was used to assess pyocyanin production by visual assessment [20]. After centrifuging the bacterial culture at 8000 rpm for 10 minutes, the absorbance of the supernatant was measured at OD_695nm_ [21]. All strains were divided into low (OD_695nm_ ≤ 1.5), moderate (OD_695nm_ of 1.5 to 3), and high (OD_695nm_ ≥ 3) pyocyanin producers. *P. aeruginosa* ATCC 27853 was used as a positive control. King A medium without any bacterial culture was used as a negative control.

### Genomic DNA Extraction

The template DNA for PCR was obtained from all *P. aeruginosa* strains using the boiling method [22]. A single colony from an overnight *P. aeruginosa* culture grown on nutrient agar was suspended in 100 μl of sterile deionised water and boiled for 10 minutes in a water bath. The suspension was then centrifuged at 13,800 rpm for 5 minutes, after which the supernatant was collected to be stored at −80 °C.

### Molecular identification of virulence-related genes

PCR was conducted to identify six virulence-associated markers: adhesion (*algD*), motility (*fliC*), T3SS (*exoS*), toxins (*plcH*, *toxR*), and quorum sensing or regulation (*lasR*) by using previously reported primers (Table 2) with minor modifications to the reaction parameters [23,24]. A Bio-Rad thermocycler (CFX96) was used for PCR amplifications using these cycling conditions: an initial denaturation at 95 °C for 15 minutes, followed by 35 cycles comprising 1 minute at 95 °C, 45 seconds at 60 °C, and 1 minute at 72 °C, with a final extension at 72 °C for 7 minutes. The PCR products were subjected to agarose (1.5%) gel electrophoresis and stained with SYBR Safe (Invitrogen) for visualisation. The gels were visualised using a UV trans-illumination system. *P. aeruginosa* ATCC 27853 was used as the positive control. A gene was considered present if a specific amplicon of expected size was visible on the agarose gel under UV illumination.

**Table 2:** Primers used in this study.

Virulence genes	Primer sequence		Amplicon size (bp)	Reference
	Forward	Reverse		
algD	AGGGCAACTGGACGGCTATC	TGTGGTCGGCAATGAAGAAGA	437	[23,24]
exoS	CTTGAAGGGACTCGACAAGG	TTCAGGTCCGCGTAGTGAAT	504	
fliC	GGCAGCTGGTTNGCCTG	GGCCTGCAGATCNCCAA	1025	
lasR	AAGTGGAAAATTGGAGTGGAG	GTAGTTGCCGACGACGATGAAG	130	
plcH	GAAGCCATGGGCTACTTCAA	AGAGTGACGAGGAGCGGTAG	307	
toxR	ATGGCATCTATGCGAGGAAC	GCAGGGGAATGAAGTTCTTG	207	
**Carbapenem genes**	**Primer sequence**		**Amplicon size (bp)**	**Reference**
	**Forward**	**Reverse**		
bla_IMP_	GGA ATA GAG TGG CTT AAY TC	TCG GTT TAA YAA AAC AAC CAC C	232	[25]
bla_VIM_	GAT GGT GTT TGG TCG CAT A	CGA ATG CGC AGC ACC AG	390	
bla_NDM_	GGT TTG GCG ATC TGG TTT TC	CGG AAT GGC TCA TCA CGA TC	621	

### PCR identification of carbapenem-resistant genes

Three carbapenemase genes (*bla*_IMP_, *bla*_VIM_ and *bla*_NDM_) (Table 2) were selected based on their prevalence in Malaysia. Identification of these genes was performed using PCR on a CFX96 Bio-Rad thermocycler as described by Nordmann et al. [25]. PCR products were analysed using 1.5% agarose gel electrophoresis and stained with SYBR Safe (Invitrogen) for identification. The gels were visualised using a UV transillumination imaging system. Amplified products were sent for DNA sequencing by Sanger Sequencing and BLAST analysis was performed using the NCBI nucleotide database using Standard Nucleotide BLAST (http://blast.ncbi.nlm.nih.gov/Blast.cgi) to validate the identity.

### Molecular typing based on MLST

Out of 215 MDR *P. aeruginosa* strains, nine strains were chosen for MLST. The selection criteria were: (i) different biofilm-forming abilities (strong, moderate and non-biofilm) and (ii) variable carbapenem-resistance levels (high, > 256 µg/mL; medium, ≥ 32 μg/mL and borderline ≥ 8 μg/mL). According to a protocol by Curran et al. [26], genes *acsA*, *aroE*, *guaA*, *mutL*, *nuoD*, *ppsA*, and *trpE* (Table 3) were selected and PCR assay was performed using a Bio-Rad thermocycler (CFX96). Single-plex PCRs were performed for each gene, starting with an initial denaturation at 96 °C for 1 minute. This was followed by 30 cycles of denaturation at 96 °C for 1 minute, primer annealing at 55 °C for 1 minute, and extension at 72 °C for 1 minute. A final extension step was conducted at 72 °C for 10 minutes. The QIAamp DNA Mini Kit (Qiagen, Hilden, Germany) was used to purify the amplicon for each target gene. DNA sequencing was carried out on an automated DNA sequencing system (Applied Biosystems, Foster City, CA). The results were compared with the data available in the PubMLST database (https://pubmlst.org/organisms/pseudomonas-aeruginosa) to determine allelic profiles and sequence types (STs). Newly identified alleles and STs were submitted to the PubMLST database for validation.

**Table 3:** Primers used for MLST [26].

Locus and function	Primer sequence (5′ to 3′)		Amplicon size (bp)
	Forward	Reverse	
*acsA*	ACCTGGTGTACGCCTCGCTGAC	GACATAGATGCCCTGCCCCTTGAT	842
*aroE*	TGGGGCTATGACTGGAAACC	TAACCCGGTTTTGTGATTCCTACA	825
*guaA*	CGGCCTCGACGTGTGGATGA	GAACGCCTGGCTGGTCTTGTGGTA	940
*mutL*	CCAGATCGCCGCCGGTGAGGTG	CAGGGTGCCATAGAGGAAGTC	940
*nuoD*	ACCGCCACCCGTACTG	TCTCGCCCATCTTGACCA	1,042
*ppsA*	GGTCGCTCGGTCAAGGTAGTGG	GGGTTCTCTTCTTCCGGCTCGTAG	989
*trpE*	GCGGCCCAGGGTCGTGAG	CCCGGCGCTTGTTGATGGTT	811

### Statistical analysis

Statistical analysis was carried out using IBM Statistical Package for Social Science (SPSS) software version 27.0 (https://www.ibm.com). The relationship between phenotypic characteristics and carbapenem resistance was assessed using the Pearson’s coefficient. For all analyses, a p-value below 0.05 was regarded as statistically significant.

## Results and discussion

### Bacterial identification and strain distribution

**Fig 1:**
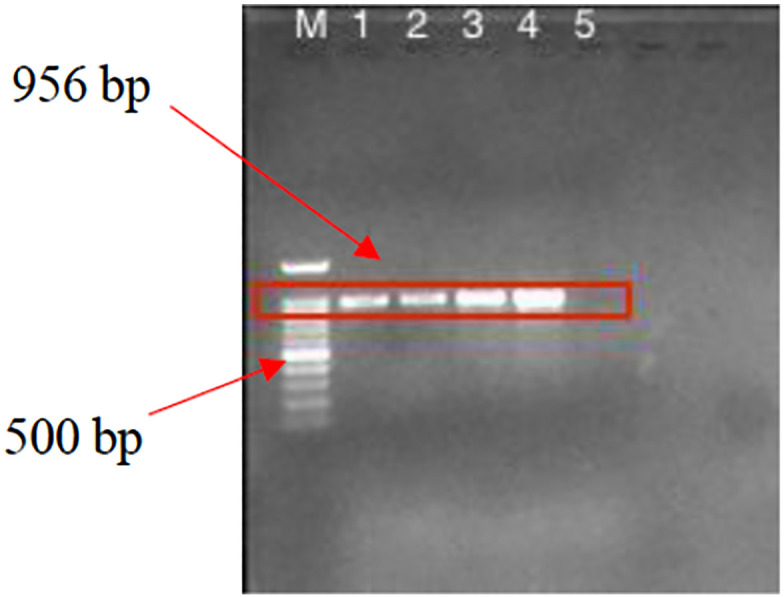
PCR-based identification of *P. aeruginosa* strains using 16S rDNA amplification. Lane M: 100 bp markers; lane 1: positive control (*P. aeruginosa* ATCC27853); lanes 2-4: clinical isolates (PACTR: lane 3: PA153; lane 4: PA205); lane 5: negative control (deionised water). The expected amplicon size is 956 bp.

Multidrug-resistant *P. aeruginosa* strains (n = 215) were collected over the three-year period from 2015 to 2018. The identity of all the strains were further verified using the PCR assay. PCR amplification targeting the 16S rDNA gene showed amplification of products with an amplicon size of 956 bp (Fig 1).

The 215 MDR *P. aeruginosa* strains were obtained from various sources across different UMMC wards. Malays were the majority race (n = 74, 34%) followed by Indians (n = 66, 31%), Chinese (n = 64, 30%) and others (n = 11, 5%). The strains were obtained from respiratory secretions (43/215, 20%), sputum (39/215, 18.1%), tissue (38/215, 17.7%) and other sources (95/215, 44.2%). Most strains were obtained from patients in the urology ward (39/215, 18.1%), intensive care unit (36/215, 16.7%), orthopaedic ward (33/215, 15.3%), neuro-intensive care unit (21/215, 9.8%), and various other wards (86/215, 40.1%). *P. aeruginosa* is a significant nosocomial pathogen, which creates major challenges due to its high rates of mortality, morbidity and the resulting healthcare expenses. While its prevalence has remained stable over the last two decades, MDR strains have increased dramatically worldwide. In Malaysia, the NSAR reported rising carbapenem resistance rates compared to 2018, with 77% of strains in this study resistant to both meropenem and imipenem [8].

### Antimicrobial susceptibility features

The antibiotic susceptibility results based on the VITEK-2 System (BioMérieux, France) showed the highest resistance rates against ceftazidime (87.4%), meropenem (80.9%) and imipenem (85.6%) (Table 4). Overall, the MDR rate was 98.6% (212/215) and 85.6% (184/215) were carbapenem-resistant strains. The MIC values for imipenem and meropenem (8 to ≥ 256 µg/mL) confirmed that 165 (76.7%) and 166 (77.2%) of the strains showed high-level resistance, respectively.

**Table 4:** Antibiotic susceptibility rates of MDR *P. aeruginosa* as determined using the VITEK-2 system.

	No. of strains, n (%)			
Antimicrobial agents	Resistant	Intermediate	Susceptible	Antibiotic class
**IPM**	184 (85.6)	0 (0)	31 (14.4)	Carbapenems
**MEM**	174 (80.9)	7 (3.3)	34 (15.8)	
**CAZ**	192 (89.3)	18 (8.4)	5 (2.3)	Cephalosporins
**FEP**	161 (74.9)	38 (17.7)	15 (7)	
**CIP**	112 (52.1)	12 (5.6)	90 (41.9)	Fluoroquinolones
**TZP**	124 (57.7)	30 (14)	61 (28.4)	Penicillins

IPM, imipenem; MEM, meropenem; CAZ, ceftazidime; FEP, cefepime; CIP, ciprofloxacin; TZP, tazobactam-piperacillin

### Phenotypic characterisation

**Fig 2:**
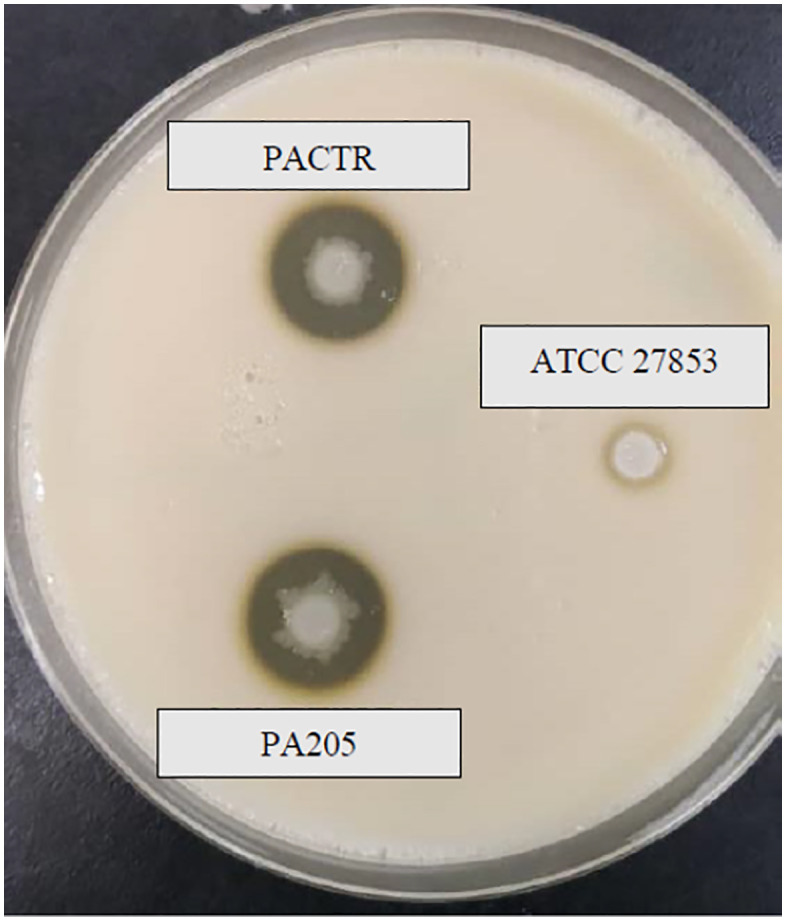
Protease activity of MDR *P. aeruginosa* strains on skim milk agar. Transparent halos indicate protease-mediated casein hydrolysis. Plates were incubated for 48 hours at 37°C. Positive control: *P. aeruginosa* ATCC 27853.

In this study, 140 (65.1%) were identified as high biofilm producers. Additionally, 47 strains (21.9%) demonstrated moderate biofilm production and 28 strains (13.0%) exhibited low biofilm production. Protease secretion was detected in 95.3% of the strains (205/215), while 4.7% (10 out of 215) could not secrete protease (Table 5). Protease secretion was detected through the transparent halo that resulted from the degradation of milk protein by protease (Fig 2). The diameter of the hydrolysis zone indicated the intensity of protease produced by the strains. *P. aeruginosa* ATCC 27853 served as the positive control.

**Table 5:** Protease production levels in MDR *P. aeruginosa* strains (n = 215) based on zone diameter on skim milk agar.

Protease assay (diameter, mm)	Frequency (n = 215)	Percentage (%)
No (0)	10	4.7
Low (≤ 1.5)	36	16.7
Medium (1.5–3.0)	71	33.0
High (≥ 3)	98	45.6

A total of 57 strains representing 26.5% showed low pyocyanin production ability. Additionally, 45 strains (20.9%) were categorised as medium producers of pyocyanin, while 113 strains (52.6%) were classified as high producers of pyocyanin (Table 6).

**Table 6:** Categorisation of MDR *P. aeruginosa* strains (n = 215) by pyocyanin production level (absorbance at OD_695nm_).

Pyocyanin assay	Frequency (n = 215)	Percentage (%)
Low	57	26.5
Medium	45	20.9
High	113	52.6

Biofilm production was observed in 92% of strains, predominantly high to moderate producers. Biofilms are critical for antibiotic resistance and persistent infections, as evidenced by prior studies [27,28]. The correlation between MBL presence and biofilm strength highlights the role of these enzymes in promoting bacterial survival under antibiotic stress, especially in hospital settings [29]. AST of the strains obtained from UMMC revealed that all strains were resistant to most of the antibiotics applied for treatment in UMMC. This test, supported by Saha et al. [30], showed that all biofilm-producing strains were highly resistant to antibiotics in comparison to the non-biofilm producers. While colistin can be effective against *P. aeruginosa*, its penetration into biofilms may be limited. Biofilm-associated infections often require a combination of antimicrobial agents and may involve the use of additional strategies, such as the use of biofilm-disrupting agents or surgical intervention [31,32]. Production of protease and biofilm were correlated, with 43% of high protease producers demonstrating moderate to high biofilm formation, consistent with findings by Zaki et al. [33]. Pyocyanin, detected in 82% of strains, further supports biofilm formation and virulence. Its interaction with extracellular DNA (eDNA) promotes bacterial aggregation, a key factor in infection persistence [34]. Phenotypic characteristics of known virulence determinants such as biofilm formation and biofilm-associated phenotypes (secretion of protease and pyocyanin) are important to understand the antibiotic resistance mechanisms that hamper treatment and control of the infection.

### Virulence gene identification and association with phenotypic traits

**Fig 3:**
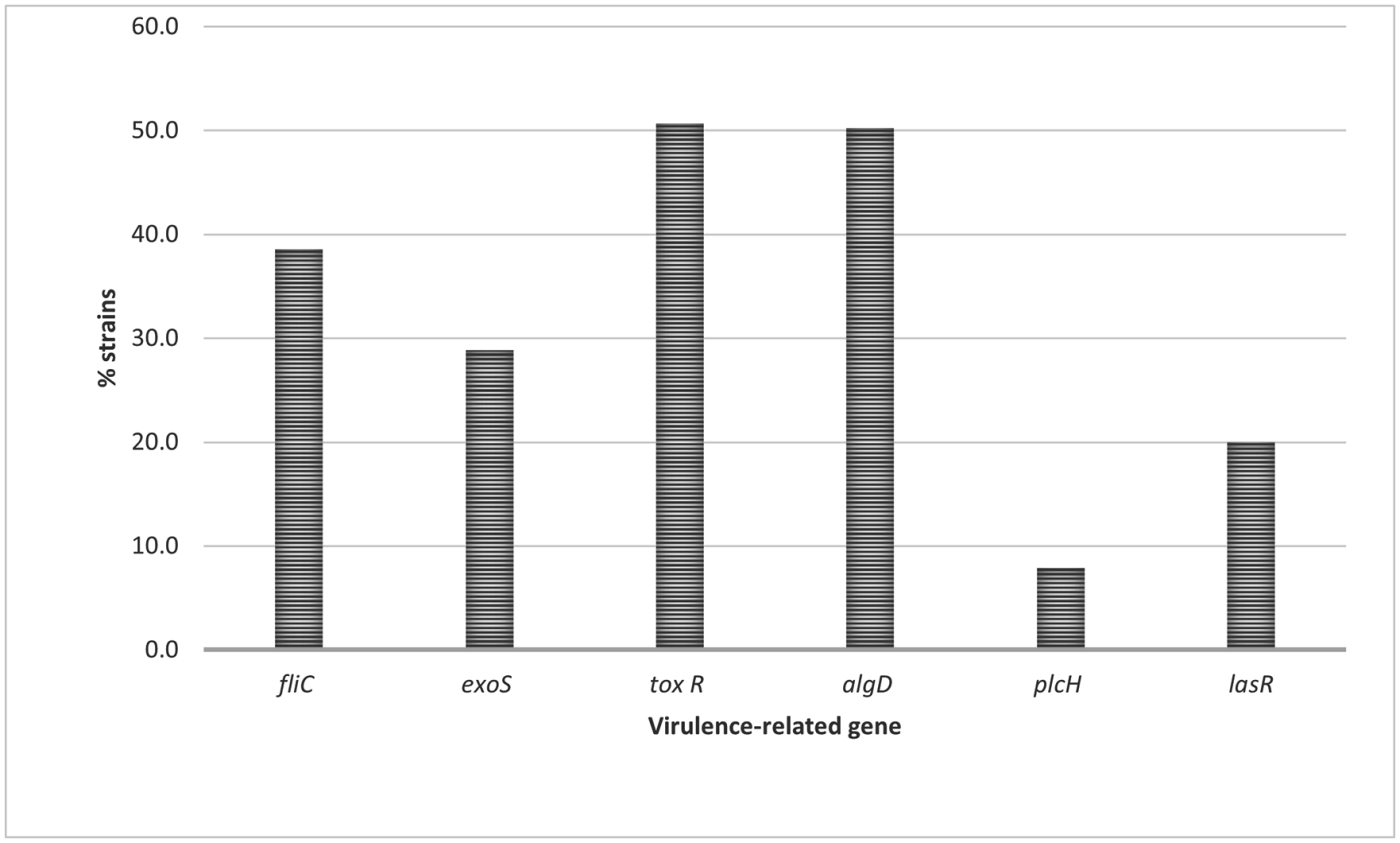
Prevalence of virulence-associated genes in the 215 MDR *P. aeruginosa* strains. Genes assessed include *fliC, exoS, toxR, algD, plcH* and *lasR.*

Among the six virulence-related genes tested, the majority of the strains harboured *toxR* (109/215, 50.7%) and *algD* (108/215, 50.2%) genes (Fig 3). The lowest identification frequencies were observed for the *plcH* (17/215, 7.9%) and *lasR* (43/215, 20.0%) genes, while all four genes *fliC, exoS, toxR* and *algD* were present in 12 of the strains. Among the total strains, 48, constituting 22%, displayed no virulence genes.

In this study, 51% of the strains carried the *toxR* gene, while 50% harboured the *algD* gene. The *toxR* in *P. aeruginosa* regulates surface-associated behaviours like swarming motility, ability to form biofilm and virulence-related factors by binding to the second messenger c-di-GMP [35]. During infection, bacteria transition from nonmucoid to mucoid phenotypes, producing alginate is essential for biofilm development [36]. Mucoid-producing bacteria become dominant in the advanced stage of infection, causing deterioration and a high mortality rate [37]. In this study, the *algD* gene was present in 50% of the strains. Several studies have investigated how virulence and antibiotic susceptibility interact, revealing an antagonistic relationship. Resistance mechanisms generate a cost to bacterial virulence [38–40]. However, other researchers proposed that the *toxR* and *algD* virulotypes are linked to carbapenem resistance, particularly in high antibiotic pressure environments, such as ICUs [41,42].

### Carbapenemase Genes: Clinical and Epidemiological Implications

Using PCR, 44 out of 215 MDR *P. aeruginosa* strains were confirmed to harbour carbapenem-resistant genes. The carbapenemase genes identified among the strains were *bla*_IMP_ (n = 24, 11.2%), followed by *bla*_NDM_ (n = 16, 7.4%) and *bla*_VIM_ (n = 4, 1.86%). Four allelic variants, *bla*_IMP-7_ (1 strain), *bla*_IMP-26_ (21 strains), *bla*_IMP-62_ (1 strain) and *bla*_IMP-76_ (1 strain), were identified. Of the 215 strains, no carbapenemase genes were detected in 190 (88.4%) strains. PA153 and PACTR showed the presence of both *bla*_IMP-26_ and *bla*_VIM-4_ genes. None of the strains had all three carbapenemase genes present. Eight, four, 14 and 18 strains from the years 2015, 2016, 2017 and 2018, respectively were positive for the carbapenemase gene (Table 7).

**Table 7:** Distribution of carbapenemase gene variants (*bla*_IMP_, *bla*_NDM_, *bla*_VIM_) in CRPA strains by year of isolation (n = 184).

Gene		CRPA strains (*n* = 184)				
		2015	2016	2017	2018	GenBank accession no.
		(*n* = 8)	(*n* = 4)	(*n* = 14)	(*n* = 18)	
*bla*_IMP_	*bla*_IMP-7_		1			AY625685.1
(*n* = 24)	*bla*_IMP-26_	7	2	5	7	NG_049190.1
	*bla*_IMP-62_		1			KX753224.1
	*bla*_IMP-76_				1	NG061409.1
*bla*_NDM_	*bla*_NDM-1_			8	8	KT364224.1
(*n* = 16)						
*bla*_VIM_	*bla*_VIM-4_	1			1	NG_050367.1
(*n* = 4)	*bla*_VIM-5_			1	1	AY456196.1

There was 98−100% DNA sequence similarity for *bla*_NDM-1_ gene for 16 *P. aeruginosa* strains studied and a *P. aeruginosa* isolate from Singapore with a GenBank accession of KT364224.1. The *bla*_VIM_ nucleotide sequence for the strains PA153 and PACTR had 100% identity to the *bla*_VIM-4_ gene with a GenBank accession of NG_050367.1 whereas PA005 and PA187 showed 99−100% identity to the *bla*_VIM-5_ gene with a GenBank accession of AY456196.1. Fourteen *P. aeruginosa* strains showed 98−100% homology to *bla*_IMP-26_ gene with a GenBank accession of NG049190.1. The GenBank accession numbers for the CRPA strains studied are shown in Table 8.

**Table 8:** GenBank accession numbers or sequenced carbapenemase genes in selected *P. aeruginosa* strains.

Strain	Gene	Accession Number
PA005	*bla*_VIM-5_	PQ574030
PA153	*bla*_VIM-4_	PQ574031
PA187	*bla*_VIM-5_	PQ574032
PACTR	*bla*_VIM-4_	PQ574033
PA040	*bla*_IMP-26_	PQ583866
PA045	*bla*_IMP-26_	PQ583867
PA065	*bla*_IMP-26_	PQ583868
PA066	*bla*_*IMP*-26_	PQ583869
PA067	*bla*_IMP-26_	PQ583870
PA080	*bla*_IMP-7_	PQ583871
PA087	*bla*_IMP-62_	PQ583872
PA088	*bla*_IMP-26_	PQ583873
PA090	*bla*_IMP-26_	PQ583874
PA105	*bla*_IMP-26_	PQ583875
PA106	*bla*_IMP-26_	PQ583876
PA108	*bla*_IMP-26_	PQ583877
PA122	*bla*_IMP-26_	PQ583878
PA124	*bla*_IMP-26_	PQ583879
PA125	*bla*_IMP-26_	PQ583880
PA153	*bla*_IMP-26_	PQ583881
PA169	*bla*_IMP-26_	PQ583882
PA170	*bla*_IMP-26_	PQ583883
PA179	*bla*_IMP-76_	PQ583884
PA181	*bla*_IMP-26_	PQ583885
PA182	*bla*_IMP-26_	PQ583886
PA189	*bla*_IMP-26_	PQ583887
PA232	*bla*_IMP-26_	PQ583888
PACTR	*bla*_IMP-26_	PQ583889
PA025	*bla*_NDM-1_	PQ583890
PA029	*bla*_NDM-1_	PQ583891
PA032	*bla*_NDM-1_	PQ583892
PA036	*bla*_NDM-1_	PQ583893
PA037	*bla*_NDM-1_	PQ583894
PA038	*bla*_NDM-1_	PQ583895
PA052	*bla*_NDM-1_	PQ583896
PA058	*bla*_NDM-1_	PQ583897
PA186	*bla*_NDM-1_	PQ583898
PA191	*bla*_NDM-1_	PQ583899
PA196	*bla*_NDM-1_	PQ583900
PA212	*bla*_NDM-1_	PQ583901
PA221	*bla*_NDM-1_	PQ583902
PA229	*bla*_NDM-1_	PQ583903
PA233	*bla*_NDM-1_	PQ583904
PA234	*bla*_NDM-1_	PQ583905

In this study, *P. aeruginosa* isolates harboured *bla*_IMP_, *bla*_NDM_ and *bla*_VIM_ carbapenemase genes, with *bla*_IMP-26_ being the most prevalent (21 out of 24 IMP-positive strains). This is consistent with prior reports from Malaysia, where *bla*_IMP-26_ was associated with endemic strains in hospital settings [43]. The study also detected strains carrying carbapenemase genes (*bla*_IMP-7,_ *bla*_IMP-62,_ *bla*_IMP-76_), albeit at low prevalence. This finding aligns with earlier reports from UMMC [44]. IMP- and VIM-type metallo-β-lactamases (MBLs) are widely reported across Asia, although prevalence varies by country and institution. In a global cohort of 972 CRPA isolates, 22% harboured carbapenemase genes [45]. In Japan, genome surveillance of 382 meropenem‐resistant isolates found a wide range of sequence types and relatively low carbapenemase prevalence [46]. In Thailand, *P. aeruginosa* isolates carrying *bla*_IMP_ and *bla*_VIM_ remain common, with ST235 reported as a major high-risk clone in national collections and outbreaks [47,48]. In China, whole-genome sequencing of CRPA strains (2018–2020) has revealed high-risk clones circulating in hospitals, highlighting the potential for horizontal spread of resistance determinants, including MBLs [49]. In India, recent work shows widespread expression of IMP and VIM MBL genes among biofilm-forming, clinical *P. aeruginosa*, suggesting a more endemic distribution than previously appreciated [50]. In the Middle East, studies from Iraq and Bahrain (2020s) report *bla*_VIM_ (especially VIM-2) and *bla*_IMP-1_ in carbapenem-resistant isolates [51]. Beyond epidemiology, there is growing concern over novel IMP variants that may evade inhibition by newer drugs (e.g., xeruborbactam) [52]. Therapeutically, promising preclinical work shows that combining imipenem with meso-dimercaptosuccinic acid (DMSA) can restore activity against MBL producers (including IMP-13 and VIM-2) in a sepsis model [53]. Together, these data indicate that while IMP- and VIM-type carbapenemases are historically well-established in Asia, especially in high-risk clones like ST235, their variant diversity is increasing, with implications for both surveillance and treatment.

The detection of rare variants like *bla*_IMP-62_ and *bla*_IMP-76_ also suggests ongoing evolution of resistance mechanisms. Other carbapenemase genes, such as *bla*_NDM-1_, linked to international transmission, highlight the role of travel in spreading resistant strains [54]. Globally, *bla*_NDM-1_, initially reported in *K. pneumoniae*, has become widespread in *P. aeruginosa*, often linked to horizontal gene transfer via plasmids [55]. Its presence in Malaysian isolates further supports the hypothesis of interspecies gene dissemination and regional mobility, possibly influenced by medical tourism and population movement [54]. The identification of *bla*_VIM-4_ and *bla*_VIM-5_, albeit in fewer isolates, remains significant due to their association with outbreaks and high-risk clones, such as ST235. These enzymes, particularly NDM and VIM types, are part of MBLs, a subclass capable of hydrolysing all β-lactams except aztreonam. They are concerning not only for their extensive resistance profiles but also for their role in promoting biofilm production, as shown by positive correlations in this study. Such dual mechanisms intensify the difficulty in treating infections and necessitate urgent containment strategies. Deeper studies are important to find out about other carbapenem resistance mechanisms.

### Novel Sequence Type ST3891: A Signpost of Genetic Diversification

MLST analysis was conducted on nine CRPA strains and identified four different STs presented in descending order by the number of strains in which the ST was found: ST235 (n = 6), ST357 (n = 1), ST882 (n = 1) and ST3891 (n = 1). PA208 was deposited in the PubMLST database on 17 February 2022, and ST3891, a novel ST, was assigned with an ID: 8202 (Table 9).

**Table 9:** Multilocus sequence typing (MLST) profiles of nine CRPA isolates based on seven housekeeping genes.

Isolate	acsA	aroE	guaA	mutL	nuoD	ppsA	trpE	ST Type
PA021	38	11	3	13	1	2	4	ST235
PA090	38	11	3	13	1	2	4	ST235
PA109	38	11	3	13	1	2	4	ST235
PA153	38	11	3	13	1	2	4	ST235
PACTR	38	11	3	13	1	2	4	ST235
PA232	38	11	3	13	1	2	4	ST235
PA148	2	4	5	3	1	6	11	ST357
PA048	17	5	11	3	4	6	37	ST882
PA208	18	4	5	3	150	17	13	ST3891

ST235 and ST357 are high-risk clones due to their MDR phenotypes [56]. In this study, ST235 was prevalent in the UMMC. A similar predominance of ST235 and ST357 in MDR *P. aeruginosa* was observed in Malaysia and other countries [54,57,58]. It is ubiquitous worldwide and linked with various resistance traits, including carbapenemases [59]. The ST235 subtype is frequently associated with poor clinical outcomes. Meanwhile, ST882 was reported in other countries but not in Malaysia [60]. The PubMLST database showed another three *P. aeruginosa* strains with ST882 were present in different locations: two strains in Australia and one strain in Russia. Three strains from different countries, including one from this study, were isolated from sputum specimens. This suggests the global distribution of this specific sequence type. A novel sequence type, ST3891, was also identified, underscoring a local evolutionary adaptation or an undetected transmission pathway within Malaysian healthcare settings. Although ST3891 was found in only one isolate, its presence alongside carbapenem resistance and virulence traits like biofilm development indicates the importance of genomic surveillance. Comparative genomics with other novel or emerging STs (e.g., ST882 found in other countries but first reported here in Malaysia) may reveal shared resistance islands or mobile genetic elements driving resistance and virulence. These findings highlight the need for continuous genetic monitoring to track the dissemination of high-risk clones.

### Correlations of phenotypic characteristics and carbapenem resistance

The results indicated that strains with high resistance to both meropenem and imipenem were also strong biofilm producers (n = 132, 61.4%). Only three carbapenem-resistant strains produced low biofilm (1.4%). The sensitivity profile of *P. aeruginosa* strains to carbapenem showed that 18 strains (8.4%) were classified as weak biofilm formers and 21 strains (9.8%) were classified as moderate biofilm formers. Significant positive correlations were observed between biofilm and protease activity (r = 0.142, p = 0.038), biofilm and pyocyanin production (r = 0.405, p < 0.001), biofilm and meropenem resistance (r = 0.729, p < 0.001), biofilm and imipenem resistance (r = 0.595, p < 0.001) and biofilm and carbapenem-resistance (r = 0.721, p < 0.001). This suggested that as the biofilm development increases, the levels of protease, pyocyanin and carbapenem-resistance tend to increase (Table 10). *P. aeruginosa* strains producing MBLs, including those with *bla*_IMP-26_ variants, demonstrated increased biofilm formation capabilities.

**Table 10:** Pearson correlation coefficients between biofilm and phenotypic characteristics of *P. aeruginosa (*n = 215).

		Biofilm	Protease	Pyocyanin	Imipenem	Meropenem	Carbapenem
Biofilm	Pearson Correlation	1.000	0.142^*^	0.405^**^	0.595^**^	0.729^**^	0.721^**^
	Sig. (2-tailed)	–	0.038	0.000	0.000	0.000	0.000
Protease	Pearson Correlation	0.142^*^	1.000	0.119	0.009	0.041	0.029
	Sig. (2-tailed)	0.038	–	0.082	0.897	0.548	0.668
Pyocyanin	Pearson Correlation	0.405^**^	0.119	1.000	0.368^**^	0.466^**^	0.460^**^
	Sig. (2-tailed)	0.000	0.082	–	0.000	0.000	0.000
Imipenem	Pearson Correlation	0.595^**^	0.009	0.368^**^	1.000	0.730^**^	0.924^**^
	Sig. (2-tailed)	0.000	0.897	0.000	–	0.000	0.000
Meropenem	Pearson Correlation	0.729^**^	0.041	0.466^**^	0.730^**^	1.000	0.910^**^
	Sig. (2-tailed)	0.000	0.548	0.000	0.000		0.000
Carbapenem	Pearson Correlation	0.721^**^	0.029	0.460^**^	0.924^**^	0.910^**^	1.000
	Sig. (2-tailed)	0.000	0.668	0.000	0.000	0.000	–

**Correlation is significant at the 0.01 level (2-tailed).

*Correlation is significant at the 0.05 level (2-tailed).

### Biofilm and virulence-related genes

Of the 215 strains categorised as high biofilm formers, 65 strains (30.2%) were found to carry the *algD* gene (biofilm associated). In contrast, low biofilm producers comprised 14/215 strains (6.5%) that harbour the *algD* gene. Among the moderate biofilm formers, 29/215 strains (13.5%) were identified to carry the *algD* gene. Most strains harboured the *toxR* (109/215, 51%) and *algD* gene (108/215, 50%). There were 83 (39%), 62 (29%) and 43 (20%) strains that exhibited *fliC*, *exoS* and *lasR* genes, respectively. The lowest identification frequencies were observed for the *plcH* gene (17/215, 8%).

### Limitations of the study

There are several limitations in this study. Firstly, it is unknown if the infections originated from environmental sources, as environmental samples were not included. This crucial information may reduce infection control implications for implementing targeted interventions that may help to prevent the spread of resistant organisms within the hospital. The current study focused on class B metallo-β-lactamases (MBLs: *bla*_IMP_, *bla*_VIM_ and *bla*_NDM_) because surveillance reports in Malaysia (NSAR 2018–2023) indicated these as the predominant carbapenemase classes. Screening for GES and KPC was not performed due to resource limitations and their historically low prevalence in local *P. aeruginosa* isolates. Knowledge on the functional relevance of these genes is also missing due to the lack of expression-level data such as quantitative PCR or transcriptomics. This limits the understanding of whether the identified genes are actively expressed and contributing to the phenotypic resistance observed. In addition, clinical correlation could not be performed due to the retrospective nature of the study and the lack of clinical metadata, such as the comorbidities and treatment outcomes. Finally, multivariate analyses were also not performed due to limitations in the dataset, including the absence of comprehensive covariate information and insufficient sample size. This restricts the ability to control for potential confounding variables and evaluate the independent contribution of each factor. Future studies with larger, well-annotated datasets, broader genomic coverage and functional validation will be crucial to improve the interpretation and clinical relevance and enable more robust interpretations.

## Conclusion

A positive correlation was found between high biofilm producers and *P. aeruginosa* strains that are resistant to carbapenem. Biofilm development may cause persistent infections and is less effective in antibiotic treatment. Based on the AST and MIC results, it can be concluded that high resistance to carbapenem may be due to the ability to form biofilm. The high biofilm formers were also supported by the result of biofilm-associated phenotype analysis, such as protease and pyocyanin assay. The clonal dissemination of ST235 in Malaysia necessitates continued close monitoring. This study presents the first report of the novel ST3891 CRPA in Malaysia. The rise of CRPA strains is alarming, highlighting the need for strict infection control policies to reduce the spread of carbapenemase-encoding genes among strains. Studies incorporating clinical and environmental strains would provide more valuable insights into the dissemination of *P. aeruginosa*.

## Supporting information

S1 File Uncropped gel and plate images of carbapenem-resistant *Pseudomonas aeruginosa* isolates. This file contains all the uncropped gel and plate images obtained in this study.
(DOCX)

S1 Table Complete dataset of 215 carbapenem-resistant *Pseudomonas aeruginosa* isolates. The dataset contains antimicrobial susceptibility, virulence profiling, biofilm formation and molecular characterisation data for all clinical isolates analysed in this study.
(XLSX)
