# Supplementary material for: The emergence of NDM-1, IMP-7, IMP-26, IMP-62, IMP-76, VIM-4 and VIM-5 producing Pseudomonas aeruginosa strains, identification of a novel sequence type (ST3891) and analysis of virulence genes from a Malaysian tertiary hospital
Source: PLoS One. 2026 Jun 10;21(6):e0350200. doi: 10.1371/journal.pone.0350200 (PMC13252801; doi:10.1371/journal.pone.0350200)
Supplement: S1 File — This file contains all the uncropped gel and plate images obtained in this study. (DOCX) [file pone.0350200.s001.docx]

**S1 File: Uncropped Gel and Plate Images**

This file contains raw, unprocessed images used to support figures in the main manuscript. Each gel or plate is labelled with sample IDs, target genes, expected band sizes and marker positions.


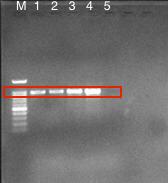


956bp

500bp

**
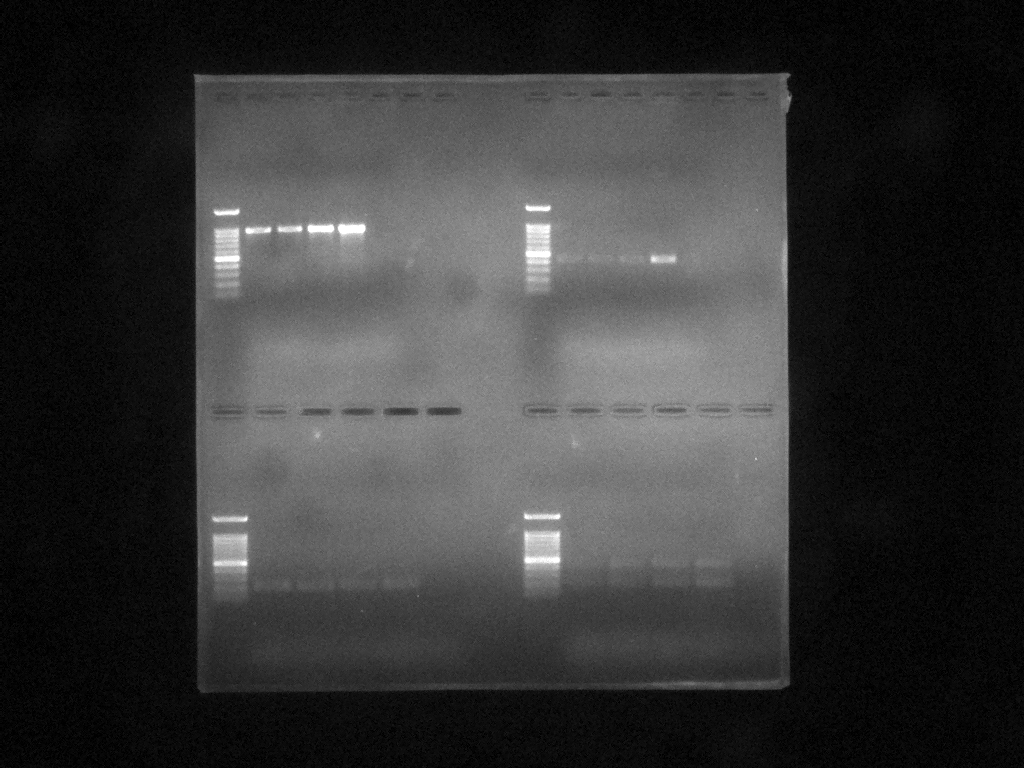
**

956bp

500bp

1 2 3 4 5 6 7 8

**Figure 1. Uncropped PCR-based identification of *P. aeruginosa* strains using 16S rDNA amplification.** Lane M: 100bp markers; lane 1: positive control (P. aeruginosa ATCC27853); lanes 2-4: clinical isolates (CTR2018-3: lane 3: PAC153; lane 4: PAC205); lane 5: negative control (deionised water). The expected amplicon size is 956bp. The three other gel parts are omitted.

**Virulence gene pcr gel**


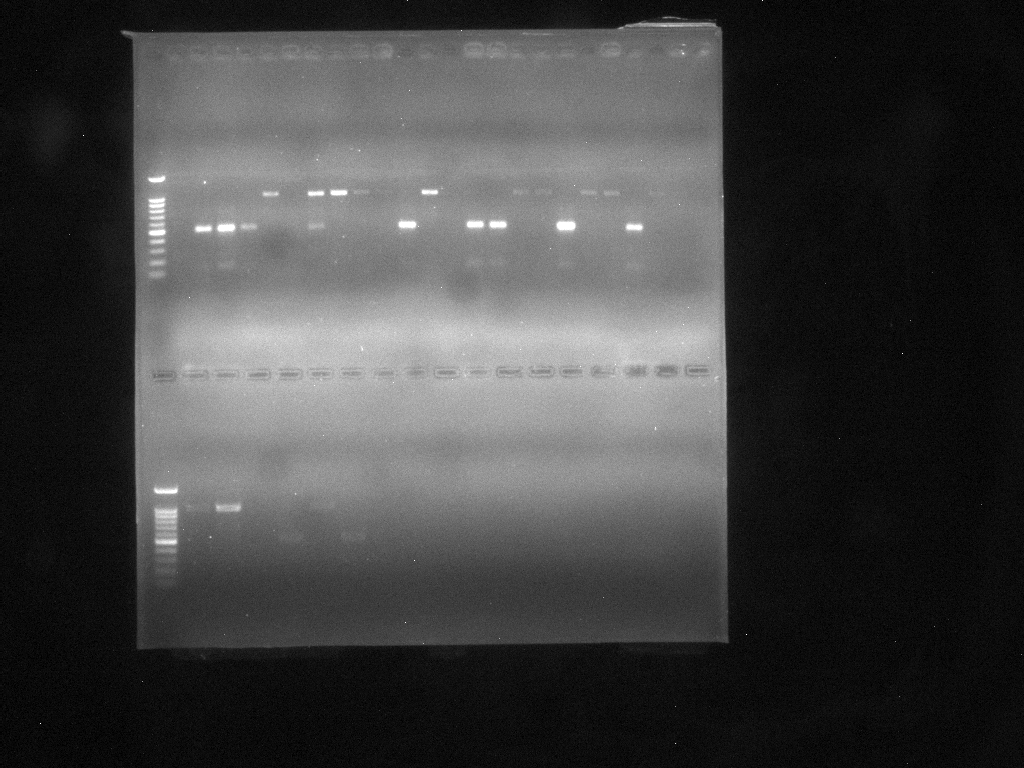


1 2 3 4 5 6 7 8 9 10 11 12 13 14 15 16 17 18 19 20 21 22 23 24 25

**1025bp**

**504bp**

**Uncropped *P. aeruginosa exoS* (504bp) and *fliC* (1025bp) identification.**

Lane 1: 100bp markers; lane 2: positive control (ATCC27853); lane 3: PAC001; lane 4: PAC002; lane 5: PAC003; lane 6: PAC004; lane 7: PAC005; lane 8: PAC006; lane 9: PAC007; lane 10: PAC021; lane 11: PAC022; lane 12: PAC023; lane 13: PAC024; lane 14: PAC025; lane 15: PAC026; lane 16: PAC027; lane 17: PAC028; lane 18: PAC029; lane 19: PAC030; lane 20: PAC031; lane 21: PAC032; lane 22: PAC033; lane 23: PAC034; lane 24: PAC035; lane 25: negative control (deionised water). No band was observed for negative control indicated that the reaction was free from contamination.


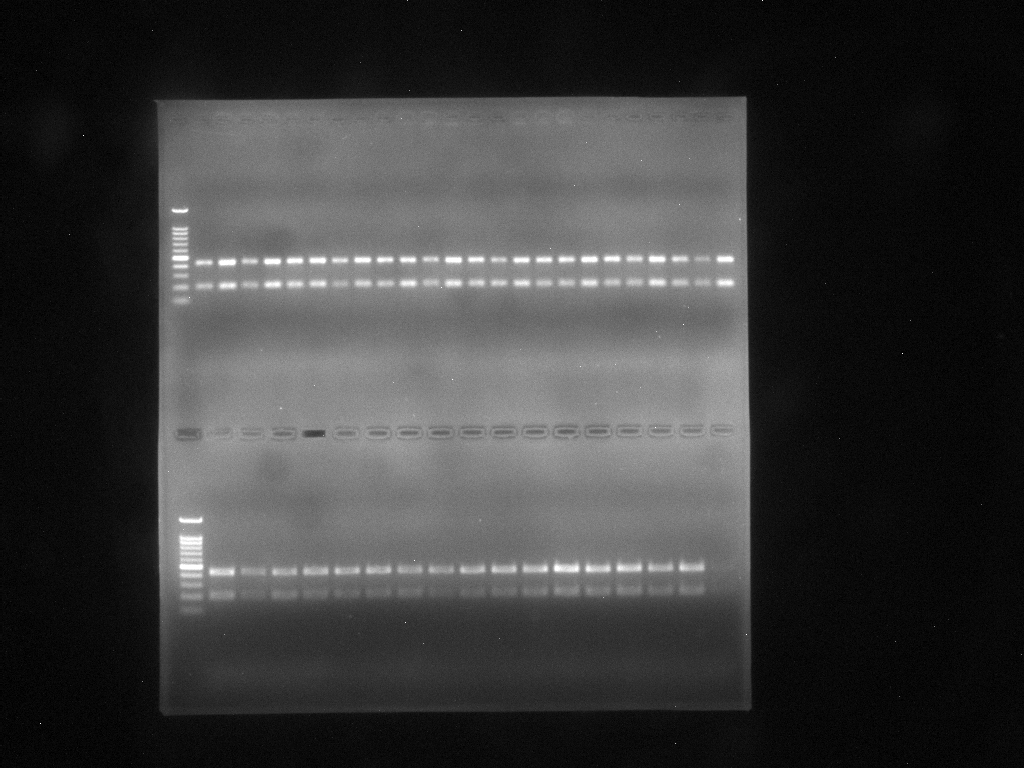


26 27 28 29 30 31 32 33 34 35 36 37 38 39 40 41 42 43

1 2 3 4 5 6 7 8 9 10 11 12 13 14 15 16 17 18 19 20 21 22 23 24 25

**207bp**

**437bp**

**Uncropped *P. aeruginosa* *toxR* (207bp) and *algD* (437bp) identification.**

Lane 1: 100bp marker; lane 2: positive control (ATCC27853); lane 3: PAC134; lane 4: PAC135; lane 5: PAC136; lane 6: PAC137; lane 7: PAC138; lane 8: PAC139; lane 9: PAC140; lane 10: PAC141; lane 11: PAC142; lane 12: PAC143; lane 13: PAC144; lane 14: PAC145; lane 15: PAC146; lane 16: PAC147; lane 17: PAC148; lane 18: PAC149; lane 19: PAC150; lane 20: PAC151; lane 21: PAC152; lane 22: PAC153; lane 23: PAC154; lane 24: PAC155; lane 25: PAC156; lane 26: 100bp marker; lane 27: PAC157; lane 28: PAC158; lane 29: PAC159; lane 30: PAC160; lane 31: PAC161; lane 32: PAC162; lane 33: PAC163; lane 34: PAC164; lane 35: PAC165; lane 36: PAC166; lane 37: PAC167; lane 38: PAC168; lane 39: PAC169; lane 40: PAC130; lane 41: PAC131; lane 42: PAC132; lane 43: negative control (deionised water). No band was observed for negative control indicated that the reaction was free from contamination.


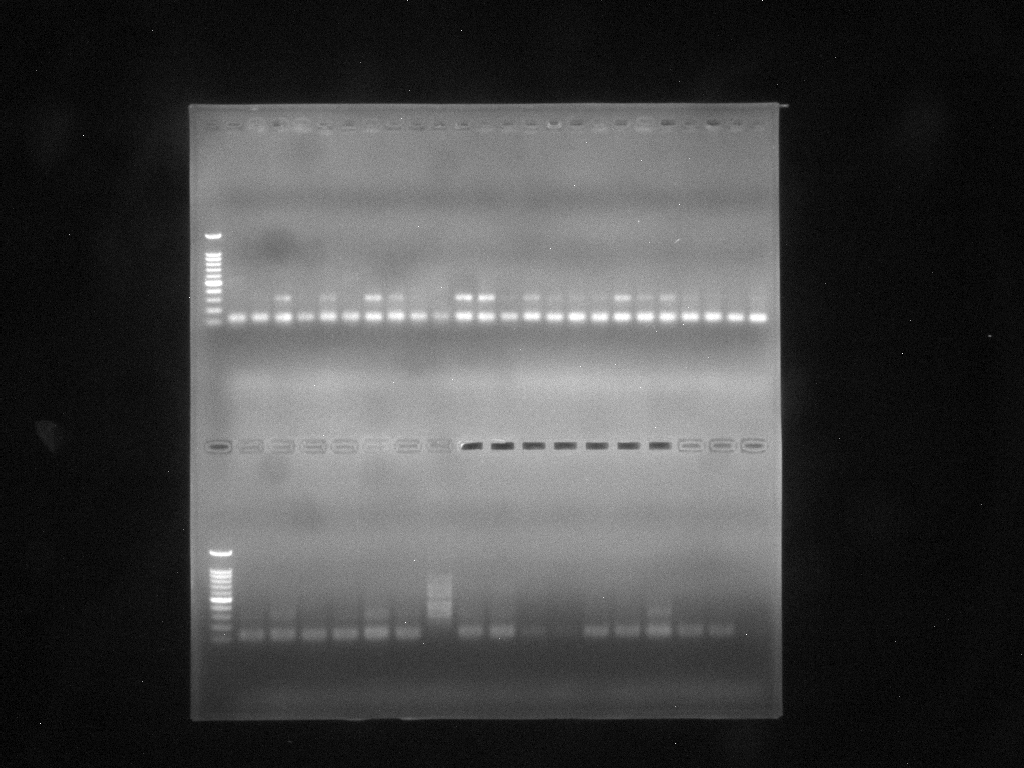


26 27 28 29 30 31 32 33 34 35 36 37 38 39 40 41 42 43

1 2 3 4 5 6 7 8 9 10 11 12 13 14 15 16 17 18 19 20 21 22 23 24 25

**130bp**

**307bp**

**Uncropped *P. aeruginosa lasR* (130bp) and *plcH* (307bp) identification.**

Lane 1: 100bp markers; lane 2: positive control (ATCC27853); lane 3: PAC001; lane 4: PAC002; lane 5: PAC003; lane 6: PAC004; lane 7: PAC005; lane 8: PAC006; lane 9: PAC007; lane 10: PAC021; lane 11: PAC022; lane 12: PAC023; lane 13: PAC024; lane 14: PAC025; lane 15: PAC026; lane 16: PAC027; lane 17: PAC028; lane 18: PAC029; lane 19: PAC030; lane 20: PAC031; lane 21: PAC032; lane 22: PAC033; lane 23: PAC034; lane 24: PAC035; lane 25: PAC036; lane 26: 100bp marker; lane 27: PAC038; lane 28: PAC039; lane 29: PAC040; lane 30: PAC041; lane 31: PAC042; lane 32: PAC043; lane 33: PAC044; lane 34: PAC045; lane 35: PAC046; lane 36: PAC047; lane 37: PAC048; lane 38:PAC049; lane 39: PAC050; lane 40: PAC051; lane 41: PAC052; lane 42: PAC053; lane 43: negative control (deionised water). No band was observed for negative control indicated that the reaction was free from contamination.

**Carbapenem gene pcr gel**


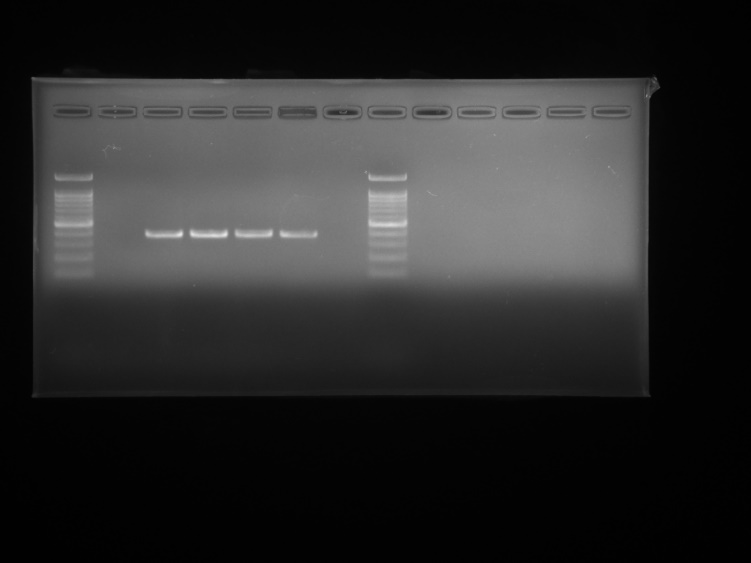


1 2 3 4 5 6 7 8

**390bp**

**Uncropped *P. aeruginosa* *bla*_VIM_ (390bp) identification.**

Lane 1: 100bp markers; lane 2: positive control (ATCC27853); lane 3: PA005: lane 4: PAC153; lane 5: PAC187; lane 6: PACTR: lane 7: negative control (deionised water); lane 8: 100bp markers. No band was observed for negative control indicated that the reaction was free from contamination.


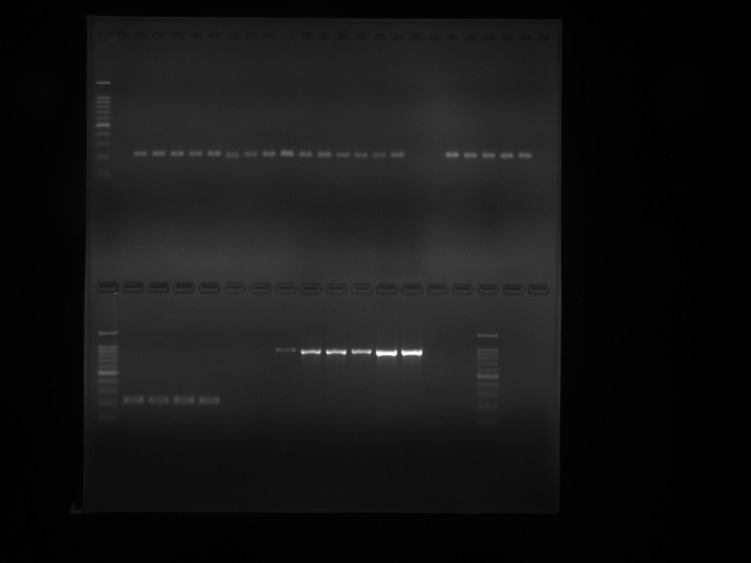


1 2 3 4 5 6 7 8 9 10 11 12 13 14 15 16 17 18 19 20 21 22 23 24 25

**232bp**

**Uncropped *P. aeruginosa* *bla*_IMP_ (232bp) identification.**

Lane 1: 100bp markers; lane 2: positive control (ATCC27853); lane 3: PAC40: lane 4: PAC45; lane 5: PAC65; lane 6: PAC66; lane 7: PAC67; lane 8: PAC80; lane 9: PAC87; lane 10: PAC88; lane 11: PAC90; lane 12: PAC105, lane 13: PAC106; lane 14: PAC108; lane 15: PAC122; lane 16: PAC124; lane 27: PAC125; lane 18: PAC129; lane 19: PAC132; lane 20: PA153; lane 21: PAC169; lane 22: PAC170; lane 23: PAC179; lane 24: PAC181; lane 25: negative control (deionised water). No band was observed for negative control indicated that the reaction was free from contamination.


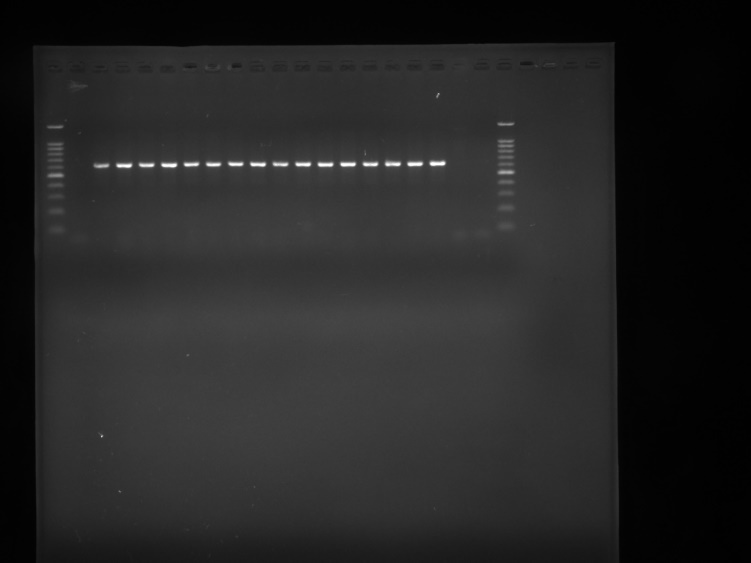


1 2 3 4 5 6 7 8 9 10 11 12 13 14 15 16 17 18 19 20 21 22 23 24 25

**621bp**

**Uncropped *P. aeruginosa* *bla*_NDM_ (621bp) identification.**

Lane 1: 100bp marker; lane 2: positive control (ATCC27853); lane 3: PAC25: lane 4: PAC29; lane 5: PAC32; lane 6: PAC36; lane 7: PAC37; lane 8: PAC38; lane 9: PAC52; lane 10: PAC58; lane 11: PAC186; lane 12: PAC191, lane 13: PAC196; lane 14: PAC212; lane 15: PAC212; lane 16: PAC221; lane 27: PAC229; lane 18: PAC233; lane 19: PAC234; lane 20: EC287; lane 21: negative control (deionised water); lane 22: 100bp marker. No band was observed for negative control indicated that the reaction was free from contamination.

**`
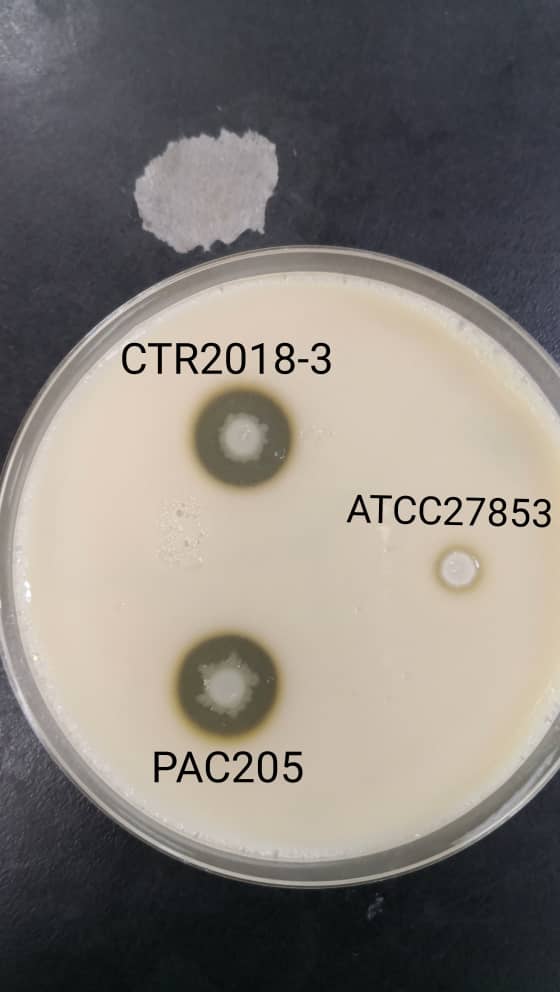
**

ATCC 27853

PA205

PACTR

**Figure 2.** **Protease assay.** Protease production by *P. aeruginosa* strains on skim milk agar. Transparent halos indicate proteolytic activity. Plates were incubated at 37°C for 48 hours. Positive control: *P. aeruginosa* ATCC 27853.

**Note: CTR2018-3 and PACTR is the same strain. Same goes to PAC205 and PA205.**
